# The Grand Challenge of Cardiovascular Epidemiology: Turning the Tide

**DOI:** 10.3389/fcvm.2014.00002

**Published:** 2014-07-30

**Authors:** Erik Ingelsson

**Affiliations:** ^1^Molecular Epidemiology and Science for Life Laboratory, Department of Medical Sciences, Uppsala University, Uppsala, Sweden

**Keywords:** cardiovascular disease, epidemiology

Cardiovascular diseases (CVD), including heart disease and stroke, comprise the global leading cause of morbidity and mortality collectively killing 12.9 million people in 2010, or one in four deaths worldwide (1). In the United States, CVD account for more than one-third of all deaths, and ~150,000 deaths per year occur in individuals younger than 65 years (2). Apart from serious consequences for the diseased individuals and their families, CVD is also associated with huge costs for society. It has been estimated that CVD and related conditions consume about a third of the total health care costs in the European Union (3). In 2010, the total costs of CVDs in the United States were estimated to be $444 billion (2). It should be noted that CVD is by no means an isolated problem for high-income countries. On the contrary, low- and middle-income countries are disproportionally affected, as more than 80% of all CVD deaths take place in such countries (4). In fact, many low-income countries are now facing a “double burden” of disease, where they continue to deal with the problems of communicable diseases and undernutrition, but at the same time they are experiencing rapidly increasing incidences of non-communicable diseases, primarily CVD and type 2 diabetes. Parts of this can be ascribed to an ongoing obesity epidemic, particularly in urban settings. According to estimations by the World Health Organization (WHO), more than 1.4 billion adults were overweight and more than 10% of the world’s adult population was obese in 2008 (5). It is not uncommon to find malnutrition and obesity existing side-by-side within the same country, same community, and even same household, and global burden from adiposity is projected to increase further in the next decade (5). Other reasons for the rapidly increasing CVD rates in low- and middle-income countries include higher exposure to risk factors, such as tobacco use, unhealthy diet, and physical inactivity; and less access to effective and equitable health care services. These factors lead to less control of hypertension, diabetes, and hyperlipidemia, and as a result, higher incidence of CVD. This rapidly increasing incidence of CVD comprises a global major challenge for public health and medical care in the decades to come (6).

Major advances in the understanding of causes and pathophysiology of CVD were made already during the 1960s and 1970s, and the widely recognized CVD risk factors, such as smoking, hypertension, hypercholesterolemia, type 2 diabetes, obesity, lack of physical activity, and family history were established before 1975 (7). In the following decades, as a result of increased understanding of the pathophysiology of ischemic heart disease, cardiac care was radically improved and major pharmaceutical drugs have contributed to dramatically decreasing case fatality rates and improved survival in patients with myocardial infarction. In contrast, similar dramatically improved survival rates have not been observed for stroke or heart failure – two other major contributors to CVD deaths. Further, although efforts to decrease cholesterol levels and smoking have been successful in the United States and Europe over the past two decades, we now see increasing prevalences of obesity and type 2 diabetes. Worrying observations include that overweight is being normalized in society (8), and that only ~1% of individuals in the United States reach what has been defined as “ideal cardiovascular health” by the American Heart Association (9, 10). In addition, the CVD incidence is now rising rapidly in developing countries as a result of adaptation to a westernized lifestyle.

So, it appears that although we know what is needed in terms of lifestyle changes to prevent CVD, the successes of implementing these in the general population are limited. For primary and primordial prevention, it is useful to distinguish between high-risk and population-wide strategies. In the former, we aim to identify the individuals at highest risk of developing disease and target them with preventive measures. An example of this is the more or less organized screening for high blood pressure and hyperlipidemia with the subsequent treatment to lower CVD risk. Further, much of the ongoing research focusing on finding new biomarkers to better stratify individuals into categories of high or low CVD risk are also aimed at improving the high-risk prevention strategy. Population-wide strategies are instead targeting the whole population to shift the risk factor distribution. The perhaps most well-known example of a successful population-wide prevention program is the North Karelia Project in Finland, where the decline in mortality from ischemic heart disease was twice as large in this region as compared to the rest of Finland between 1969 and 1979 (24 vs. 12% in men; 51 vs. 24% in women) (11). However, this and several other examples of successful prevention programs in the community notwithstanding, the global trends of increasing burden from CVD and increasing risk factors give us a clear signal. New strategies for control of CVD risk factors and prevention of CVD in the community are desperately needed, and this is not only one of the grand challenges of cardiovascular epidemiology, but poses as one of the central threats to global public health calling for urgent action.

Furthermore, even if there are a few exceptions like ticagrelor (platelet aggregation inhibitor), rosuvastatin (second-generation statin), and rivaroxaban (oral anticoagulant); the pharmaceutical industry at large has been unsuccessful in developing new drugs for primary and secondary prevention of CVD in the past decade. Hence, there is a large need for revitalization of the cardiovascular field with: (a) improved risk prediction and more adequate individually tailored treatment; and (b) new targets for drug development based on pathways previously unknown to be involved in CVD pathophysiology.

Great progress has been made in defining the genetic architecture of CVD and associated conditions over the past 5 years as a result of the development of high-throughput genotyping technology and its main application; genome-wide association studies (GWAS). This knowledge has provided many new clues to disease pathophysiology through implication of biological pathways previously not hypothesized to be involved in the development of disease. There are already good examples of how new important biology has been discovered using this technique (12), but the full impact of GWAS findings is yet to be explored through follow-up studies. In parallel with the rapid development of genotyping using microarray techniques and bioinformatic analyses of single nucleotide polymorphisms (SNPs) from such platforms, the technological development in next generation sequencing methods has been dramatic, by far exceeding Moore’s law (that predicts a doubling of computing power every 2 years). From January 2008 to the present day, the cost for a high-coverage whole-genome scan has decreased from 10 million USD to 1,000 USD – the “1000-dollar genome” can now be achieved using the Illumina HiSeq X Ten platform. Sequencing platforms can be used for a range of applications, including DNA sequencing to map causal variants and explore rare variation, RNA sequencing for expression analyses, and methylation sequencing for epigenetic studies. Other large-scale omics techniques that are currently undergoing a rapid development include proteomics and metabolomics using mass spectrometry, nuclear magnetic resonance spectroscopy, or affinity-based techniques. The full scope of these methods has still not been appreciated, but with the rapidly decreasing costs and increasing throughput, we are now able to start implementing them in large population-based study samples to disentangle the mechanisms leading to CVD.

Standing at the brink of this omics revolution, it is quite clear that the opportunities for exploration of new pathways leading to CVD and other complex diseases using these methods are previously unseen, and that the full power will be reached by combining data from all of these methods. By studies through the whole chain of intermediate steps, DNA → mRNA → protein → metabolites → subclinical CVD → overt CVD, and the complex interactions between parts of these pathways, we can improve understanding of CVD pathophysiology to identify new drug targets, as well as novel biomarkers for risk stratification and treatment decisions.

The grand challenge of cardiovascular epidemiology is to turn the tide of increasing CVD morbidity and mortality globally. To do this, we will need new tools for better risk stratification to maximize efficiency of high-risk strategies for prevention; new approaches to population-wide prevention; and new knowledge about the mechanisms leading to CVD to provide leads for development of a new generation of pharmaceutical drugs. As the Charles Dickens novel “A Tale of Two Cities” begins “*It was the best of times, it was the worst of times, it was the age of wisdom*,” I believe that cardiovascular epidemiologists are indeed living in a challenging time with increasing burden from CVD and limited success in primary prevention, but at the same time in the best of times with the rapid developments of new tools for biomedical discoveries. It is the goal of *Frontiers in Cardiovascular Medicine* to provide a forum for presenting creative research and new approaches to meet the challenges of our time. We hope that you want to take us up on the grand challenges of cardiovascular epidemiology, and help us turning the tide.

## Conflict of Interest Statement

The author declares that the research was conducted in the absence of any commercial or financial relationships that could be construed as a potential conflict of interest.

## References

[1] Lozano R Naghavi M Foreman K Lim S Shibuya K Aboyans V Global and regional mortality from 235 causes of death for 20 age groups in 1990 and 2010: a systematic analysis for the Global Burden of Disease Study 2010. Lancet (2012). 380(9859):2095–128. 10.1016/S0140-6736(12)61728-0 23245604 PMC10790329

[2] Centers for Disease Control and Prevention. Chronic Disease Prevention and Health Promotion. Available from: http://www.cdc.gov/chronicdisease/resources/publications/aag/dhdsp.htm

[3] Leal J Luengo-Fernandez R Gray A Petersen S Rayner M. Economic burden of cardiovascular diseases in the enlarged European Union. Eur Heart J (2006) 27(13):1610–9. 10.1093/eurheartj/ehi733 16495286

[4] World Health Organization. Fact Sheet on Cardiovascular Diseases. Available from: http://www.who.int/mediacentre/factsheets/fs317/en/

[5] World Health Organization. Fact Sheet on Obesity and Overweight. Available from: http://www.who.int/mediacentre/factsheets/fs311/en/

[6] World Health Association. The World Health Report 2002: Risks to Health 2002. Geneva: World Health Association (2002).

[7] Kannel WB. Some lessons in cardiovascular epidemiology from Framingham. Am J Cardiol (1976) 37(2):269–82. 10.1016/0002-9149(76)90323-4 1246956

[8] Lundahl A Kidwell KM Nelson TD. Parental underestimates of child weight: a meta-analysis. Pediatrics (2014) 133(3):e689–703. 10.1542/peds.2013-2690 24488736

[9] Yang Q Cogswell ME Flanders WD Hong Y Zhang Z Loustalot F Trends in cardiovascular health metrics and associations with all-cause and CVD mortality among US adults. JAMA (2012) 307(12):1273–83. 10.1001/jama.2012.339 22427615 PMC9004324

[10] Ford ES Greenlund KJ Hong Y. Ideal cardiovascular health and mortality from all causes and diseases of the circulatory system among adults in the United States. Circulation (2012) 125(8):987–95. 10.1161/CIRCULATIONAHA.111.049122 22291126 PMC4556343

[11] Salonen JT Puska P Kottke TE Tuomilehto J Nissinen A. Decline in mortality from coronary heart disease in Finland from 1969 to 1979. Br Med J (1983) 286(6381):1857–60. 10.1136/bmj.286.6381.1857 6407602 PMC1547760

[12] Lander ES Initial impact of the sequencing of the human genome. Nature (2011) 470(7333):187–97. 10.1038/nature09792 21307931

